# Multinomial models of the repetition-based truth effect: Investigating the role of prior knowledge

**DOI:** 10.3758/s13421-025-01821-x

**Published:** 2026-01-28

**Authors:** Oliver Schmidt, Daniel W. Heck

**Affiliations:** Department of Psychology, Philipps-Universität Marburg, Gutenbergstraße 18, Room 01059, Marburg, D-35037 Germany

**Keywords:** Repetition-based truth effect, Receiver operating characteristics (ROC), Multinomial processing tree (MPT) models, Knowledge conditional model, Fluency conditional model

## Abstract

The repetition-based truth effect refers to the phenomenon that repeated statements are more likely to be judged as true than new statements. Fazio et al. (*Journal of Experimental Psychology: General,* *144*(5), 993–1002, 2015) developed two multinomial processing tree (MPT) models to account for truth judgments. The knowledge-conditional model assumes that repetition leads to a shift in response bias conditional on the absence of knowledge. In contrast, the fluency-conditional model assumes that knowledge is used only when not relying on processing fluency, which results in reduced discrimination performance. We study the formal properties of the competing models using receiver operating characteristic (ROC) curves and highlight important auxiliary assumptions and identifiability constraints. In three experiments, we extended the classic truth-effect paradigm to validate and test different model versions by manipulating the base rate of true statements in the judgment phase. The results support the notion that repetition results in reduced discrimination performance. However, the alternative model conceptualizing the truth effect as a response bias cannot be rejected when assuming different knowledge for true and false statements.

## Introduction

The repetition-based truth effect refers to the robust phenomenon that repeated statements are more likely judged to be true than novel ones (Hasher et al., 1977). As discussed in recent reviews and meta-analyses (Dechêne et al., 2010; Unkelbach et al., 2019; Henderson et al., 2022), researchers have examined several cognitive processes that contribute to illusory truth, such as recognition (Bacon, 1979; Hawkins & Hoch, 1992), familiarity (Arkes et al., 1989), and coherence (Unkelbach & Rom, 2017). According to fluency theories, statements are more likely judged as true if they can be easily processed (Reber & Schwarz, 1999). Many studies have tested this prediction by manipulating processing fluency in various ways (for overviews, see Alter and Oppenheimer, 2009; Brashier and Marsh, 2020; Unkelbach and Greifeneder, 2013). While the effect of repetition has turned out to be very robust Dechêne et al. 2010, other manipulations of processing fluency, such as varying the visual contrast of presented statements, have yielded mixed results (Aktepe & Heck, 2025a).

It is commonly assumed that the occurrence and size of the truth effect depend on a person’s knowledge about the validity of statements (Dechêne et al., 2010; Parks & Toth, 2006). Based on the assumption that the truth effect should be largest for ambiguous statements, studies have often used difficult statements for which participants likely have no or only limited knowledge (e.g., Hasher et al., 1977; Unkelbach, 2007; Boehm, 1994). An example of an unknown, true statement is “Starfish have no brains,” whereas an example of a known, false statement is “Lyon is the capital of France.” However, recent research has shown that the repetition-based truth effect also occurs if participants have substantial or even certain knowledge (e.g., Fazio et al., 2015; Fazio et al., 2019; Fazio, 2020; Lacassagne et al., 2022), in turn questioning the moderating role of prior knowledge (Fazio et al., 2019; but see Aktepe and Heck, 2025b).

Fazio et al. (2015) developed two competing multinomial processing tree (MPT) models to disentangle the contributions of fluency and knowledge to the truth effect. The first model assumes that people rely on repetition-induced fluency only conditional on the absence of knowledge, reflecting the common assumption that a lack of knowledge is necessary for the truth effect to occur (Dechêne et al., 2010). In contrast, the second model assumes that people generally rely on processing fluency as a cue and only retrieve knowledge if fluency is not used to make a judgment. The empirical results of Fazio et al. (2015) supported the latter model, indicating that participants sometimes neglect their knowledge, implying that “knowledge does not protect against illusory truth.”

The article by Fazio et al. (2015) has had a large impact, as indicated by more than 800 citations. However, the models have not yet been validated systematically, a common requirement for MPT models (Schmidt et al., 2025). The lack of direct empirical validation studies is especially problematic for the interpretation of the model parameters. While both models assume that the model parameter *F* (introduced in the next section) reflects only processing fluency, it may also measure the contributions of other repetition-related processes such as familiarity, recognition, or coherence (Unkelbach et al., 2019). In addition, we are aware of only a few (successful) replication studies testing the multinomial models (Calio et al., 2020; Fazio and Sherry, 2020) or supporting their core assumptions (Fazio, 2020).

Besides replicating and extending the findings by Fazio et al. (2015), we elaborate on auxiliary assumptions and predictions of the models.

In the following, we first introduce the two competing MPT models of the truth effect. Next, we discuss the model predictions in terms of implied receiver operating characteristics (ROC) curves. We show that the models make opposing predictions about whether repetition results merely in a response bias towards giving more “true” responses or in a decreased performance of providing accurate truth judgments. We also show that the former model is equivalent to the two-high-threshold model (2HTM) for truth judgments (Hilbig, 2012). Next, we discuss issues concerning model specifications and parameter constraints to shed light on the question of which auxiliary assumptions are necessary and which may be problematic for plausibility and model identifiability. We empirically test the competing MPT models in three experiments by manipulating the base rate of true statements. By studying whether different base rates selectively influence the guessing parameter, we provide a stronger test of validity compared to simpler designs (Fazio et al., 2015).

### Multinomial models of the repetition-based truth effect

MPT models are statistical models for discrete data that allow disentangling and measuring the contributions of different latent cognitive processes to observable behavior (for a tutorial and a review, see Schmidt et al., 2025, and Erdfelder et al., 2009, respectively). The latent cognitive processes of interest (e.g., whether people rely on knowledge) are represented by model parameters in terms of conditional probabilities in a probability tree. MPT models of the repetition-based truth effect make predictions regarding the observed frequencies of “true” and “false” responses for different experimental conditions (e.g., statements that are repeated & true, repeated & false, new & false, etc.). A major advantage of MPT models is the requirement to precisely formalize the underlying assumptions of a theory, which facilitates explicit tests of model predictions and model comparisons.

Several MPT models have been developed for the repeti-tion-based truth effect. For instance, Unkelbach and Stahl (2009) developed an MPT model for the effect of source credibility on truth judgments, while Nadarevic and Erdfelder (2025) modeled different memory states underlying the repetition-based truth effect.

Since we are interested in the truth effect conditional on or upon the absence of prior knowledge, we focus only on the two MPT models developed by Fazio et al. (2015), each of which assumes that three cognitive processes are relevant for judging truth, namely, knowledge, fluency, and guessing.

**Fig. 1:**
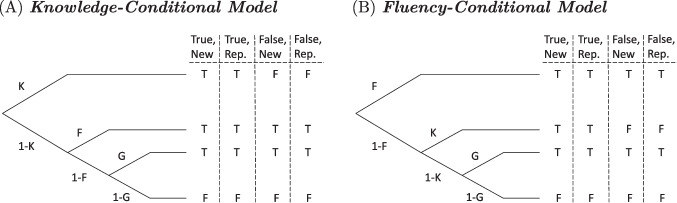
Multinomial processing tree models of the truth effect by Fazio et al. (2015)

The knowledge-conditional model (KCM) assumes that people initially search their memory for stored knowledge and only use fluency as a cue for truth judgments if the search has been unsuccessful (Fazio et al., 2015). Figure 1A shows that knowledge is retrieved from memory with probability *K*, and in turn, the truth status of the statement is accurately judged. Conditional on the absence of knowledge, people rely on fluency with probability *F* in which case they always judge a statement to be “true.” This event has the joint probability $(1-K) \cdot F$. In contrast, with probability $1-F$, people do not rely on fluency. Given that people neither retrieve knowledge nor rely on fluency, they guess that a statement is true with probability *G*, an event that occurs with the joint probability $(1-K) \cdot (1-F) \cdot G$.

In contrast to the KCM, the fluency-conditional model (FCM) assumes a different order of conditional probabilities for the latent cognitive processes (see Fig. 1B). The model predicts that truth judgments are based on fluency first, and only if this is not the case, knowledge is retrieved. Hence, with probability *F*, participants rely on fluency, meaning that they always judge a statement as “true” irrespective of its actual truth status. With probability $1-F$, participants do not rely on fluency and retrieve knowledge stored in memory with probability *K*. If this succeeds, they give an accurate response with the joint probability $(1-F)\cdot K$. If participants neither rely on fluency nor retrieve knowledge, they guess that a statement is true with probability *G*.

Importantly, the interpretation of the *F* parameter as representing processing fluency is an assumption that currently lacks empirical validation. According to the structure of both MPT models, the *F* parameter may not exclusively capture fluency but also other cognitive processes affected by repetition (e.g., familiarity, recognition, coherence, or a combination thereof; Silva et al., 2017; Unkelbach, 2007). In the following, we retain the original label *F* for this parameter to ensure consistency with Fazio et al. (2015), but we avoid an interpretation of *F* as a process-pure measure of fluency. Instead, we focus on the core question of whether the effect of repetition is conditional on the absence of knowledge or not.

Although both models assume that three latent cognitive processes are relevant for truth judgments, they crucially differ concerning the assumed structure and order of conditional probabilities. The predicted response probabilities illustrate the differences between the two models. According to the KCM, the probability that people judge true and false statements to be “true” is

1 $$\begin{aligned} P_\text {KCM}(\text {``true''} \mid \text {true})&= K + (1-K)\cdot F +(1-K)\cdot (1-F)\cdot G \nonumber \\ P_\text {KCM}(\text {``true''} \mid \text {false})&= (1-K)\cdot F +(1-K)\cdot (1-F)\cdot G. \end{aligned}$$

In contrast, the FCM assumes the following conditional probabilities:

2 $$\begin{aligned} P_\text {FCM}(\text {``true''} \mid \text {true})&= F + (1-F) \cdot K + (1-F)\cdot (1-K)\cdot G \nonumber \\ P_\text {FCM}(\text {``true''} \mid \text {false})&= F + (1-F)\cdot (1-K)\cdot G. \end{aligned}$$

Fazio et al. (2015) tested the KCM and the FCM in a $2 \times 2 \times 2$ truth-effect experiment with the factors repetition, knowledge, and truth status. Knowledge was manipulated by selecting two sets of statements categorized as “known” or “unknown” based on knowledge norms and multiple-choice questions at the end of the study. Manipulating knowledge is necessary to render the models in Fig. 1 identifiable (see Section “Model specification: Identifiability and auxiliary assumptions”). Fazio et al. (2015) parameterized the models for the three-factorial design using five model parameters. More precisely, they assumed separate parameters representing knowledge for known ($K_{k}$) and unknown ($K_{u}$) statements and fluency for new ($F_{n}$) and repeated statements ($F_{r}$), and a single guessing parameter (*G*) across all conditions. Overall, this renders both the KCM and the FCM testable with $df = 8-5 = 3$ degrees of freedom.

In Experiment 2 of Fazio et al. (2015), the FCM fitted the data well, whereas the KCM did not. This implies that repetition affected participants’ truth judgments unconditionally of knowing a statement’s truth status. Fazio et al. (2015) concluded that repetition-induced fluency is used as the dominant cue for truth judgments. Even though *F* may reflect different cognitive processes, the conclusion that repetition has an effect unconditionally on the use of knowledge holds. Calio et al. (2020) provided further support for the better fit of the FCM compared to the KCM in a laboratory experiment, which examined the effect of warning instructions on the truth effect. Overall, previous studies supported the FCM over the KCM, providing evidence for the assumption that repetition unconditionally affects truth judgments even when knowledge is available (see also Fazio & Sherry, 2020; Lacassagne et al., 2022).

**Fig. 2:**
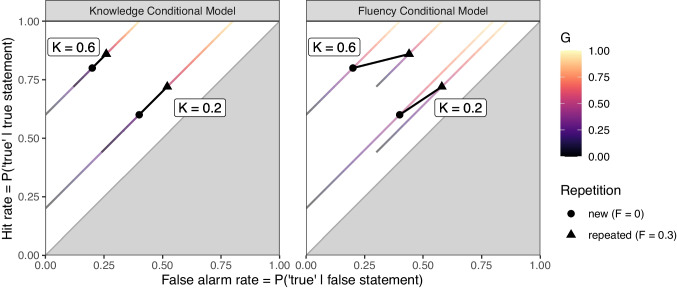
Illustration of model predictions in ROC space. The *black points* and *lines* refer to the model predictions for a standard $2\times 2$ experimental design assuming a fixed guessing probability of $G =.50$. The colored iso-sensitivity curves are derived by varying the parameter *G* from zero to one

### Receiver operating characteristic (ROC) curves

To gain a deeper understanding of the implications of the FCM and KCM, we focus on receiver operating characteristic (ROC) curves for truth judgments. ROC curves are a visual tool for assessing discrimination performance in binary classification tasks (for a primer, see Hautus et al., 2021) and are frequently used in recognition memory experiments (e.g., Bröder & Schütz, 2009). However, their application in the context of truth judgments is relatively uncommon (for an exception, see Hilbig, 2012). ROC plots allow us to visualize and assess model predictions, thereby providing a more comprehensive understanding of the competing models and their underlying assumptions. Thereby, it will become evident that the KCM and the FCM offer distinct explanations of whether the observed truth effect merely represents a shift in response bias or results in a decrease in the overall discrimination performance.

Figure 2 illustrates ROC plots for truth judgments. The *x*-axis represents the *false-alarm rate* defined as the probability of mistakenly judging a false statement to be “true.” The *y*-axis represents the *hit rate* defined as the probability of correctly judging a true statement to be “true”. Accordingly, optimal performance manifests as the point (0, 1) in the top left corner, whereas chance-level performance corresponds to all points on the main diagonal from (0, 0) to (1, 1). ROC plots often show observed or predicted response probabilities for different experimental conditions via separate points, each corresponding to a combination of a hit and a false-alarm rate. In such a plot, ROC curves are obtained by connecting those points that correspond to a certain level of knowledge. Such lines are also referred to as iso-sensitivity curves because they illustrate how hit and false-alarm rates change when only the latent decision threshold for giving a “true” or “false” judgment changes while knowledge is held constant. For a given ROC or iso-sensitivity curve, the area under the curve (AUC) serves as a metric that quantifies the overall performance of a participant or a model, with a greater AUC indicating superior accuracy.

For a better understanding of the different assumptions of the KCM and FCM, it is valuable to plot their predictions in ROC space. One can derive the coordinates of a single point by defining specific values for the parameters *K*, *F*, and *G* and computing the predicted hit and false-alarm rate. For the standard $2 \times 2 \times 2$ design with the factors knowledge, repetition, and truth status, the last factor (i.e., whether a statement is true or false) is implicitly represented by the *x*- and *y*-axis of the ROC space. The predictions of the two models can thus be visualized by four points for the remaining factors, knowledge and repetition. The four points (shown in black) are derived under the assumption that the guessing parameter *G* remains constant. The colored lines in Fig. 2 illustrate how varying the guessing parameter *G* from zero to one (represented by colors from violet to yellow) provides model-implied ROC curves.

**Fig. 3:**
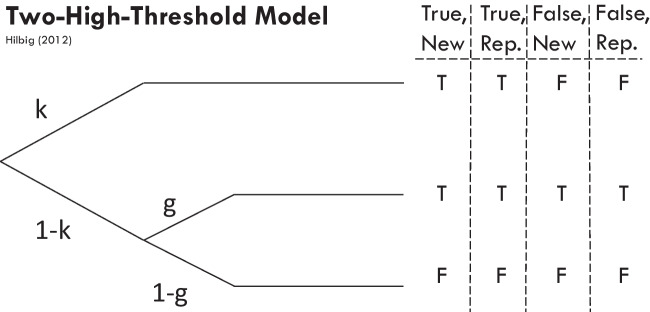
The two-high-threshold model (2HTM) of truth judgments by Hilbig (2012). The parameters refer to knowledge (*k*) and guessing (*g*). The original 2HTM has not yet been applied in the context of the repetition-based truth effect

The KCM postulates that the effect of repetition manifests as an increased likelihood of responding “true” conditional on a lack of knowledge. This implies that the unconditional probability of relying on knowledge is identical for new and repeated statements. Repetition is assumed to lead to a larger *F* parameter, and hence, an increased tendency to respond “true” in the absence of knowledge. As shown in Fig. 2, the hit and false-alarm rates for new and repeated statements are assumed to lie on the same iso-sensitivity curve, which can be derived from the model equations in Eq. 1 as

3 $$\begin{aligned} P(\text {Hit}) = K + P(\text {FA}). \end{aligned}$$

In the ROC space in Fig. 2, repetition results in a proportional increase in false alarms and hits (i.e., the predicted points “move” towards the upper right corner). This implies that the truth effect does not result in a change in overall accuracy, as the AUC remains constant. Overall, the KCM assumes that the repetition-based truth effect emerges due to a shift in response bias. This assumption is in line with previous conceptualizations of the truth effect as a response bias (e.g., Unkelbach, 2007).

By assuming that repetition merely affects response bias, the KCM represents a special case of the two-high-threshold model (2HTM) of truth judgments (Hilbig, 2012). Hilbig (2012) proposed and validated the 2HTM to investigate the framing effect, according to which negatively framed statements are more likely to be considered true. For example, the statement “20% of marriages are divorced within the first 10 years” is more likely judged as true compared to the equivalent, but positively framed statement “80% of marriages last 10 years or longer.” The model disentangles the contributions of knowledge *k* and, conditional on the absence of knowledge, response bias *g* on truth judgments (see Fig. 3). From the perspective of the 2HTM, the truth effect merely presents a certain type of response bias that results in a larger *g* parameter. This implies that the false-alarm and hit rates for new and repeated statements predicted by the KCM are all on the same ROC curve (i.e., the iso-sensitivity curve shown in colors from violet to yellow in Fig. 2). The KCM only differs from the 2HTM in making additional assumptions about the relative size of the guessing parameter *g* across the $2\times 2$ conditions involving the factors knowledge and repetition (see Section “Model specification: Identifiability and auxiliary assumptions”).

The FCM assumes that repetition generally leads to an increase in the unconditional probability *F* of judging statements to be “true.” Since knowledge only becomes relevant with probability $(1-F)$, it is less frequently used for repeated than for new statements. Consequently, for repeated statements, the model predicts a decrease in the discrimination performance. In Fig. 2, this is reflected by a shift of the false-alarm and the hit rate towards chance level (i.e., towards the diagonal) within the ROC space. From the perspective of the 2HTM, the shift predicted by the FCM implies that judgments for new and repeated statements are located on different iso-sensitivity curves, which can be derived from the model equations in Eq. 2 as

4 $$\begin{aligned} P(\text {Hit}) = (1-F) \cdot K + P(\text {FA}). \end{aligned}$$

A derivation of ROC curves for the FCM can be found in the Appendix.

While both the KCM and the FCM incorporate a parameter *F* that increases the likelihood of responding “true” for repeated statements, their theoretical implications differ regarding the source and consequences of this effect. In the KCM, repetition only affects truth judgments conditionally on the absence of knowledge. That is, repetition affects response tendencies given that no knowledge is retrieved, which is conceptually similar to the notion of a conditional response bias, as in the 2HTM. Importantly, this kind of bias does not affect the probability of relying on one’s knowledge and thus preserves the overall discrimination performance of differentiating true from false statements (as reflected by an unchanged AUC). In contrast, the FCM defines the *F* parameter as an unconditional probability to respond “true” for repeated statements, regardless of whether knowledge is available. Thus, reliance on repetition-related cues such as fluency or familiarity overrides existing knowledge. This mechanism reduces the probability that participants rely on their knowledge, thereby impairing overall discrimination performance. In the ROC space in Fig. 2, the effect of repetition is visually represented by a proportionally larger increase in the false-alarm rate than in the hit rate. This results in a smaller AUC for repeated than for new statements. Hence, although both models contain a repetition-related *F* parameter, only the KCM justifies interpreting it as a (performance-irrelevant) response bias in the narrow sense, whereas the FCM links it to a reduced discrimination performance for truth judgments due to a shift toward chance level.

### Model specification: Identifiability and auxiliary assumptions

Model identifiability refers to a model’s property that, given a specific data set, one always obtains the same unique values for the parameter estimates. This is an important requirement for interpreting the parameters of MPT models (Schmidt et al., 2025). Technically, a model is identifiable if the parameter vector $\boldsymbol{\theta }$ generates unique predictions for the predicted response probabilities (i.e., a one-to-one mapping; Bamber and van Santen, 2000). For the KCM and the FCM, this means that different values of the parameter vector $\boldsymbol{\theta }=(K, F, G)$ result in different predicted probabilities in terms of the model Eqs. 1 and 2, respectively. If a model is globally identified, unique parameter estimates can be obtained for all possible parameter vectors $\boldsymbol{\theta }$. In contrast, local identifiability is a weaker condition and ensures only the uniqueness of parameter estimates in the neighborhood of a specific parameter vector $\boldsymbol{\theta }^*$. For instance, regarding the KCM, the parameters *F* and *G* can only be estimated if the parameter *K* differs from one, that is, if participants do not have perfect knowledge.

The original versions of the KCM and the FCM by Fazio et al. (2015) for the $2\times 2 \times 2$ design (with the factors knowledge, repetition, and truth status) both make parameter constraints that result in $\text {df}=3$ degrees of freedom. Specifically, both models assume that (a) *K* only differs between unknown and known statements, (b) *F* only differs between new and repeated statements, and (c) *G* is identical for all statements. Whereas these assumptions are intuitive and substantively plausible, they result in a critical issue. Even though the degrees of freedom are strictly positive, the KCM lacks identifiability and cannot provide unique parameter estimates. The lack of model identifiability can be shown by repeatedly analyzing the same dataset. Although the same response frequencies are fitted multiple times, the estimated KCM parameters vary arbitrarily between repetitions. This implies that one cannot interpret the parameter estimates obtained for a specific data set.

A closer look at the differences between the 2HTM and the KCM explains the lack of identifiability of the KCM. Both models share similarities and assume that the truth effect is due to a response bias. However, unlike the KCM, an adapted version of the 2HTM with separate guessing probabilities for each of the $2\times 2$ conditions (i.e., known vs. unknown crossed with new vs. repeated) maintains model identifiability (Hilbig, 2012; Heck & Erdfelder, 2016). The essence of the identifiability problem in the KCM is due to the specific parameterization of response tendencies conditional on the absence of knowledge. The KCM defines three parameters $F_{r}$, $F_{n}$, and *G* which determine the probability of responding “true” in the absence of knowledge. At first glance, it is surprising that the 2HTM has more guessing parameters (i.e., four *g* parameters for the $2\times 2$ conditions) but is still identifiable. To resolve this apparent contradiction, we link the models formally by defining the four guessing parameters of the 2HTM as a function of the three parameters of the KCM,

$$\begin{aligned} g_{k, r}&= F_\text {r} + (1-F_{r})\cdot G \\ g_{k, n}&= F_\text {n} + (1-F_{n})\cdot G\\ g_{u, r}&= F_\text {r} + (1-F_{r})\cdot G \\ g_{u, n}&= F_\text {n} + (1-F_{n})\cdot G. \end{aligned}$$

All four *g* parameters of the 2HTM are identifiable by the data, which means that a separate response bias can be estimated for the four conditions shown in Fig. 2 (Heck & Erdfelder, 2016). However, even though we have four unique values on the *left* side of the equation system, it is impossible to obtain a unique solution for the three parameters $F_{r}$, $F_{n}$, and *G* of the KCM. This is because there are only two unique equations on the *right* side (i.e., the last two equations are identical to the first two, which means that the model makes the same predictions). Technically, the duplicate equations result in a non-full rank of the Jacobian matrix of the KCM, which implies that the model is not identifiable (Schmidt et al., 2025). Intuitively, the issue can also be seen in the tree diagram for the KCM in Fig. 1. Conditional on a lack of knowledge, the second and the third branch, which include the parameters *F* and *G*, respectively, both result in the same predicted response patterns (i.e., four times “true”).

To render the KCM identifiable, an effective solution involves adding further equality constraints on the model parameters. Reducing the number of free parameters that need to be estimated ensures the uniqueness and improves the precision of parameter estimates. A lack of identifiability implies that different constraints can lead to the same model fit. Hence, selecting appropriate constraints requires careful consideration. For the KCM, we propose adding theoretically interpretable constraints by setting the *F* parameter associated with new statements to zero (i.e., $F_{n}=0$). This constraint, already used by Calio et al. (2020) for convenience, is based on the notion that the parameter *F* does not play any role in processing new statements. Instead, only the parameter $F_{r}$ matters because it encodes the *relative increase* in judging *repeated* statements as ‘true’ conditional on the absence of knowledge, as predicted by higher fluency, familiarity, or related processes. From the perspective of the 2HTM, the constraint $F_{n}=0$ results in the order constraint that $g_{r}$ has to be larger than $g_{n}$ (Knapp & Batchelder, 2004). From a substantive perspective, the constraint thus aligns with the conceptualization of the repetition-based truth effect as an upward shift in response bias (i.e., an increased tendency to respond “true” to repeated statements). Overall, the new version of the KCM not only addresses the identifiability issue but also maintains the theoretical interpretation of the KCM.

In contrast to the KCM, the specification of two *F* parameters does not pose a challenge for the FCM, as the remaining latent processes are defined conditional on not relying on repetition. The *F* parameter is specified as an unconditional probability, which mitigates concerns related to non-identifiability of the *F* parameters for repeated and new statements. Still, to facilitate a fair model comparison between the two competing models, we introduce a new model variant of the FCM model that implements the same equality constraint $F_{new} = 0$ as the KCM. This adjustment ensures that both models have the same number of parameters, thus serving as a baseline for evaluating model performance across different conditions.

It is noteworthy that the FCM lacks local identifiability if $K_{k} = K_{u}$. This is due to the assumption of the FCM that repetition already results in decreased overall discrimination performance for truth judgments. Technically, the lack of identifiability can be seen when considering the predicted response probabilities in Fig. 1B conditional on not relying on fluency or a combination of fluency-related processes (denoted by $\lnot F$),

$$\begin{aligned} P_\text {FCM}(\text {``true''} \mid \text {known, true, } \lnot F)&= K_* + (1-K_*)\cdot G \\ P_\text {FCM}(\text {``true''} \mid \text {known, false, } \lnot F)&= (1-K_*)\cdot G\\ P_\text {FCM}(\text {``true''} \mid \text {unknown, true, } \lnot F)&= K_* + (1-K_*)\cdot G \\ P_\text {FCM}(\text {``true''} \mid \text {unknown, false, } \lnot F)&= (1-K_*)\cdot G. \end{aligned}$$

Even though there are four unique equations for the data on the left side, the last two equations are again duplicates of the first two equations whenever there is only a single knowledge parameter $K_*=K_{k}=K_{u}$. The lack of local identifiability is not a critical issue. However, it emphasizes that researchers testing the FCM in empirical studies should select statements that clearly differ in knowledge, as this ensures that $K_{k}$ is notably larger than $K_{u}$. Put differently, the experimental manipulation of knowledge should be sufficiently strong to estimate the model parameters of the FCM reliably and to facilitate high informativeness of model comparisons with the KCM.

Both the KCM and the FCM make the auxiliary assumption that individuals have equal knowledge of true and false statements, as indicated by the implicit constraint $K_{t} = K_{f}$. This assumption will usually be unproblematic for unknown statements for which knowledge is negligible anyway (i.e., the *K* parameter will be close to zero both for true and false statements). However, the auxiliary assumption can be crucial for known statements because participants might have different levels of knowledge about false and true statements. The equal-knowledge assumption is obviously problematic when using separate item sets for true and false statements. In terms of experimental design, the issue may partially be addressed by selecting well-calibrated item materials. However, even when creating a true and a false version of each statement, the difficulty of the false version might not match that of the true version. For instance, it is easy to see that the statement “The Mediterranean Ocean is the largest ocean on Earth” is wrong, whereas more errors are expected when referring to the Atlantic Ocean.

The validity of the auxiliary assumption of equal knowledge for true and false statements is particularly important since its violation might contribute to a bad performance of the KCM, as empirically observed by Fazio et al. (2015). Different levels of knowledge for true and false statements would result in a steeper or shallower slope of the ROC curve for known statements in Fig. 2A (Hilbig, 2012). This could result in insufficient model fit of the KCM, whereas the FCM can (partially) compensate for varying slopes via the *F* parameters for new and repeated statements. A simple solution to this issue is to fit the KCM with separate parameters $K_{t}$ and $K_{f}$ for true and false statements in each knowledge condition. A reanalysis of the openly available data by Fazio et al. (2015) shows that an extended version of the KCM with different knowledge parameters fits well ($G^2(2) = 1.06$, $p = 0.590$),^1^ indicating more knowledge for known true than for known false statements ($K_{k,t} = 0.681$, 95% CI: [.548, .814]; $K_{k,f} =.262$, 95% CI: [.048, .475]) but similar knowledge for unknown statements ($K_{u,t} =.000$, 95% CI: [0, .401]; $K_{u,f} = 0.135$, 95% CI: [0, .383]). The reanalysis provides preliminary evidence for the importance of allowing different knowledge parameters for true and false statements in the KCM.

**Table 1:** Parameter constraints of different model variants for a $2\times 2\times 2\times 2$ design

	KCM_equal_	KCM_unequal_	FCM_nonzero_	FCM_zero_
	$(\text {df} = 10)$	$(\text {df} = 8)$	$(\text {df} = 8)$	$(\text {df} = 10)$
Fluency	$F_{n,30} = F_{n,70} = 0$	$F_{n,30} = F_{n,70} = 0$	$F_{n,30}$	$F_{n,30} = F_{n,70} = 0$
			$$F_{n,70}$$	
	$F_{r,30}$	$F_{r,30}$	$F_{r,30}$	$F_{r,30}$
	$F_{r,70}$	$F_{r,70}$	$F_{r,70}$	$F_{r,70}$
Guessing	$G_{30}$	$G_{30}$	$G_{30}$	$G_{30}$
	$G_{70}$	$G_{70}$	$G_{70}$	$G_{70}$
Knowledge	$K_{k,t} = K_{k,f}$	$K_{k,t}$	$K_{k,t} = K_{k,f}$	$K_{k,t} = K_{k,f}$
		$$K_{k,f}$$		
	$K_{u,t} = K_{u,f}$	$K_{u,t}$	$K_{u,t} = K_{u,f}$	$K_{u,t} = K_{u,f}$
		$$K_{u,f}$$		

*Note.* The constraints correspond to an experimental design with the between-subject factor base rate (70% vs. 30%) and the within-subject factors knowledge (known, unknown), repetition (repeated, new), and truth status (true, false)

### Model validation via base-rate manipulations

For the empirical validation of the KCM and the FCM, we extend the standard experimental design by Fazio et al. (2015) by manipulating the base rate of true statements (e.g., 30% vs. 70%) in a $2 \times 2 \times 2 \times 2$ mixed design with base rate as a between-subjects factor. By adopting this approach, which is often applied for the 2HTM and in recognition-memory research in general (e.g., Malejka & Bröder, 2019), our experiments shed light on the joint effects of base rates and repetition on response tendencies in truth judgments.

The manipulation of base rates serves three goals. First, our experiments aim at validating the KCM and FCM by testing whether base rates selectively influence the guessing parameter *G*. We adopt the common assumption that different base rates cannot influence knowledge (*K*), while participants may be more or less inclined to respond “true” when having to guess (Malejka & Bröder, 2019). Second, the base-rate manipulation allows us to fit an extended version of the KCM with different knowledge parameters for true and false statements. Thereby, we can relax a critical auxiliary assumption that might have contributed to the poor performance of the KCM in prior studies (Fazio et al., 2015; Calio et al., 2020). Third, the extension with base rates provides a more severe test of the KCM against the FCM compared to prior studies. By extending the design, the models need to account for a more complex data structure, and in turn, the likelihood of an informative test between the competing models increases.

Extending the KCM and FCM to a $2 \times 2 \times 2 \times 2$ mixed design requires additional assumptions regarding parameter constraints. The manipulation of base rates is expected to affect the probability *G* of guessing “true,” which is captured by separate parameters for the two conditions (i.e., $G_{30}$ and $G_{70}$). In contrast, the impact of base rates on the truth effect and the parameter *F* remains uncertain. On the one hand, one could assume that base rates *selectively* influence the guessing probability *G* and, as a consequence, should thus have no effects on the parameter *F*. This is based on the assumption that repeated statements are already encoded at prior exposure before the base rate is known. On the other hand, one could also argue that base rates shape the context for judging the truth of repeated statements (see Unkelbach & Greifeneder, 2013). Hence, the effect of repetition, and thus the parameter *F*, might differ between conditions. To test whether base rates selectively influence the probability to guess “true” and “false”, we initially assume separate *F* parameters for the two base-rate conditions (i.e., $F_{n,30}$ and $F_{n,70}$ for new statements and $F_{r,30}$ and $F_{r,70}$ for repeated statements). Selective influence can then be assessed by testing the hypotheses $F_{n,30}=F_{n,70}$ and $F_{r,30}=F_{r,70}$.

Furthermore, it is implausible for base-rate manipulations to influence knowledge. Therefore, the parameter *K* must be constant across different base-rate conditions. Irrespective of this invariance assumption, the *K* parameter can still vary for known versus unknown and true versus false statements in some model versions. Our general assumptions serve as a basis for a nuanced exploration of response tendencies in the context of base-rate manipulations.

By incorporating additional parameter constraints, we obtain the four model versions shown in Table 1. Each model allows us to test different auxiliary assumptions. According to the original KCM by Fazio et al. (2015), the knowledge parameter is assumed to be equal for true and false statements (i.e., $K_{k,t} = K_{k,f}$ and $K_{u,t} = K_{u,f}$ for known and unknown statements, respectively). As explained in the previous section, model identifiability is ensured by assuming that the fluency parameter for new statements is zero ($F_{n,30} = F_{n,70} = 0$). Consequently, the model, referred to as the KCM_equal_, has $\text {df} = 10$ degrees of freedom. The model KCM_unequal_ addresses the potentially problematic assumption of invariant knowledge for true and false statements. This adapted version allows for different knowledge parameters for the factor truth status. As such, the KCM_unequal_ includes four knowledge parameters ($K_{k,t}$, $K_{k,f}$, $K_{u,t}$, $K_{u,f}$). Given the constraint on the fluency parameters for new statements ($F_{n,30} = F_{n,70} = 0$), the extended KCM has $\text {df}=8$ degrees of freedom.

Regarding the FCM, we adopted the original FCM by Fazio et al. (2015) for the $2 \times 2 \times 2 \times 2$ mixed design. The FCM_nonzero_ model is similar to the original model version and allows all four fluency parameters ($F_{n,30}$, $F_{n,70}$, $F_{r,30}$, $F_{r,70}$) to be freely estimated. Moreover, the knowledge parameter differs only between known and unknown statements ($K_{k,t}=K_{k,f}$ and $K_{u,t}=K_{u,f}$). Thereby, the FCM_nonzero_ model has $\text {df} = 8$ degrees of freedom. To facilitate a fair model comparison between the KCM and the FCM, we also fitted the FCM_zero_ version, which implements the same parameter restrictions as the KCM_equal_. This version differs from the original FCM of Fazio et al. (2015) in the additional assumption that the *F* parameter is zero for new statements ($F_{n,30} = F_{n,70} = 0$). Thus, fluency and related processes are assumed to be relevant only for repeated statements. However, the FCM_zero_ still assumes identical knowledge for true and false statements ($K_{k,t} = K_{k,f}$ and $K_{u,t} =K_{u,f}$).

Despite the gain in information provided by the base-rate manipulation, identifying a version of the FCM with separate parameters $K_{t}$ and $K_{f}$ is challenging. This is due to the assumption that fluency already affects the overall performance of discrimination for truth judgments, as indicated by distinct iso-sensitivity curves with varying slopes in Fig. 2. Essentially, it is impossible to disentangle whether differences in discrimination performance are due to different knowledge of true and false statements or due to different fluency of new and repeated statements. However, we consider the extension of the FCM for varying knowledge to be less important than the extension of the KCM, since the former model fit well, whereas the latter did not (Fazio et al., 2015; Calio et al., 2020).

Fazio et al. (2015) fitted their models based on response frequencies aggregated across individuals, a common appro-ach in MPT modeling (Schmidt et al., 2025). However, the analysis of aggregated response frequencies in within-subject designs can cause statistical issues if individuals differ in parameters. As a remedy, we rely on both classic, frequentist MPT models for aggregated data and hierarchical Bayesian MPT models (Heck et al., 2018). We fit separate hierarchical models for the (between-subjects) base-rate conditions while modeling the effects of the within-subject factors on the parameters using different parameter labels.

We impose the constraint that the *F* parameter for new statements is zero ($F_{n}=0$). Moreover, the hierarchical MPT models include parameters for the effect of repetition due to fluency and related processes ($F_{r}$), guessing tendencies (*G*), and knowledge for known and unknown statements ($K_{k,t} = K_{k,f}$ and $K_{u,t} = K_{u,f}$). These constraints render the hierarchical MPT models similar to the models KCM_equal_ and FCM_zero_ in Table 1. However, the models are not exactly identical since they allow for different knowledge parameters *K* for the two base-rate conditions. We also fit a version of the KCM with different *K* parameters for true and false statements, which resembles the model KCM_unequal_ with separate *K* parameters for the two base-rate conditions.

## Experiment 1

Experiment 1 tests whether the truth effect manifests either as a response bias, as posited by the KCM, or as a reduced performance of discriminating between true and false statements, as suggested by the FCM. To address this question, we conducted an online experiment extending the classic truth-effect paradigm by manipulating the base rate of “true” statements to be either 70% or 30%. Participants did not receive any monetary incentives for correct judgments.

**Table 2:** Mean percentages of true judgments

Base rate	Knowledge	Status	Repetition	Study 1	Study 2	Study 3
30%	known	true	new	.709 [.660, .757]	.758 [.719, .797]	.759 [.725, .793]
30%	known	true	repeated	.752 [.703, .801]	.809 [.774, .843]	.784 [.747, .820]
30%	known	false	new	.152 [.119, .186]	.171 [.140, .201]	.192 [.163, .220]
30%	known	false	repeated	.254 [.201, .307]	.209 [.177, .240]	.253 [.208, .297]
30%	unknown	true	new	.422 [.374, .469]	.516 [.473, .559]	.479 [.437, .521]
30%	unknown	true	repeated	.526 [.461, .592]	.562 [.514, .610]	.520 [.479, .562]
30%	unknown	false	new	.320 [.285, .355]	.392 [.358, .426]	.374 [.342, .406]
30%	unknown	false	repeated	.418 [.372, .463]	.442 [.407, .477]	.424 [.388, .461]
70%	known	true	new	.733 [.694, .772]	.831 [.806, .856]	.808 [.786, .830]
70%	known	true	repeated	.772 [.735, .810]	.871 [.849, .893]	.874 [.851, .897]
70%	known	false	new	.186 [.138, .233]	.269 [.225, .313]	.292 [.249, .335]
70%	known	false	repeated	.293 [.234, .352]	.386 [.331, .440]	.439 [.385, .493]
70%	unknown	true	new	.485 [.439, .530]	.629 [.593, .664]	.624 [.592, .655]
70%	unknown	true	repeated	.578 [.528, .628]	.736 [.701, .771]	.745 [.713, .778]
70%	unknown	false	new	.368 [.318, .418]	.504 [.462, .547]	.522 [.482, .563]
70%	unknown	false	repeated	.505 [.447, .563]	.620 [.574, .666]	.636 [.586, .686]

*Note.* Percentages of true judgments were first computed for each person and then aggregated. In Study 3, the base rates communicated to participants were 25% and 75%

### Methods

#### Participants

We based our sample size on established conventions in prior research on the truth effect (cf. Schmidt & Heck, 2024; Henderson et al., 2022) and recruited 236 participants via the professional panel provider *bilendi*. We excluded eight participants who did not complete the entire study, 13 participants who indicated that they did not work on the study seriously, and seven participants who did not speak German as their native language. To ensure high data quality, we excluded 16 participants because they rated 25% of all statements either extremely fast (< 1 s) or extremely slow (> 30 s), and five participants who completed the experiment extremely fast (total time < 10 min). Furthermore, 17 participants had to be excluded due to technical issues that prevented the recording of response times. At the trial level, we also excluded extremely fast (< 1 s) and slow (> 30 s) truth judgments. Based on these criteria, 134 judgments were removed. These thresholds were based on pretests, assuming that responses under 1 s barely enabled participants to read and process a statement, whereas responses exceeding 30 s increased the likelihood that participants looked up statements. The final sample included $N = 170$ participants (84 male, 86 female) with a mean age of 51.9 ($SD = 16.4$, ranging from 18 to 82) and 71.21 truth judgments on average.

#### Design & materials

We extended the truth-effect paradigm by manipulating the base rate of true statements as a between-subjects factor (70% vs. 30% true statements). Thus, we implemented a $2 \times 2 \times 2 \times 2$ mixed design with the three within-subjects factors repetition (new vs. repeated), knowledge (known vs. unknown), and truth status (true vs. false). We used 96 trivia statements by Schmidt and Heck (2024), which are available in the supplementary materials. Based on the accuracy of dichotomous responses in the previous study, 48 statements were categorized as known ($M =.89$, $SD =.05$) and unknown ($M =.52$, $SD =.01$) each (where a probability of $M =.50$ refers to chance level).

#### Procedure

The online experiment was conducted using the software SoSci Survey (Leiner, 2019). The project file, which includes all instructions, is available at https://osf.io/c86wq/. After receiving general information about the experiment and providing informed consent and demographic information, participants were randomly assigned to one of the two base-rate conditions. The exposure phase asked participants to rate their interest in 36 trivia statements, which were randomly selected while counterbalancing the within-subject factors knowledge and truth status. A base rate of either 30% or 70% of the presented statements was true, while this information was not communicated to the participants. Following two example statements, participants rated their interest in the 36 statements on a scale from 1 (“very uninteresting”) to 6 (“very interesting”). Statements were presented one at a time and were rated in a self-paced manner. Next, participants completed an intermediate, unrelated puzzle as a distractor task.

In the judgment phase, participants were informed that the base rate of true statements was either 70% or 30%. In line with this information, participants rated 72 statements, of which either 48 or 24 were true, respectively (i.e., 12 or six true statements in each of the four factorial combinations of truth status and knowledge). Participants were instructed to rate the truth of each statement and were informed that they might have encountered some of the statements in the previous phase of the experiment. Before starting the actual judgment phase, participants saw two example statements. Next, participants provided binary truth judgments (“true” or “false”) for 72 statements. Each statement was presented on a separate screen. The base rate of true statements was highlighted by a permanent box in either green (70%) or red (30%) above each presented statement to remind participants of the base rate. The colors green and red were selected due to their ecological correlation with true and false information (Nadarevic et al., 2022). At the end, participants received feedback on the percentage of correct judgments. Eventually, participants stated whether they had worked seriously on the study, were thanked, and debriefed.

**Table 3:** Model fit indices based on aggregated frequencies

**Experiment 1**					
Model version	df	$G^2$	*p*	AIC	BIC
KCM_equal_	10	12.10	.278	24.10	68.51
KCM_unequal_	8	7.91	.443	23.91	83.12
FCM_nonzero_	8	9.17	.239	25.17	84.38
FCM_zero_	10	9.17	.517	**21.17**	**65.57**
**Experiment 2**					
Model version	df	$G^2$	*p*	AIC	BIC
KCM_equal_	10	25.32	.005	37.32	82.97
KCM_unequal_	8	18.87	.016	34.87	95.74
FCM_nonzero_	8	13.14	.107	29.14	90.00
FCM_zero_	10	14.84	.138	**26.84**	**72.49**
**Experiment 3**					
Model version	df	$G^2$	*p*	AIC	BIC
KCM_equal_	10	41.34	<.001	53.34	99.87
KCM_unequal_	8	16.79	.032	32.79	94.82
FCM_nonzero_	8	8.79	.361	24.79	86.83
FCM_zero_	10	11.31	.334	**23.21**	**69.84**

*Note.* Fitted model versions reflect different auxiliary assumptions for the knowledge-conditional model (KCM) and the fluency-conditional model (FCM). The degrees of freedom (*df*), the likelihood ratio test statistic $G^2$, its *p*-value, and the information criteria AIC and BIC are shown

**Table 4:** Parameter estimates of MPT models fitted to aggregated response frequencies

Parameter	Model variant			
	KCM_equal_	KCM_unequal_	FCM_nonzero_	FCM_zero_
	$\text {df} = 10$	$\text {df} = 8$	$\text {df} = 8$	$\text {df} = 10$
**Experiment 1**				
$F_{n,30}$	0	0	.000 [.000; .048]	0
$F_{n,70}$			.000 [.000; .063]	
$F_{r,30}$	.200 [.153; .248]	.177 [.120; .234]	.127 [.080; .174]	.127 [.095; .158]
$F_{r,70}$	.212 [.162; .262]	.201 [.149; .252]	.158 [.098; .218]	.158 [.119; .197]
$G_{30}$	.349 [.326; .372]	.305 [.234; .375]	.358 [.312; .405]	.358 [.336; .381]
$G_{70}$	.420 [.396; .443]	.368 [.284; .452]	.419 [.367; .472]	.419 [.396; .443]
$K_{k,f}$	.521 [.449; .543]	.449 [.334; .564]	.556 [.526; .585]	.556 [.532 ;.579]
$K_{k,t}$		.569 [.506; .632]		
$K_{u,f}$	.102 [.076; .128]	.000 [.000; .200]	.113 [.083; .142]	.113 [.085; .141]
$K_{u,t}$		.174 [.061; .284]		
**Experiment 2**				
$F_{n,30}$	0	0	.000 [.000; .042]	0
$F_{n,70}$			.039 [.000; .100]	
$F_{r,30}$	.120 [.069; .171]	.104 [.056; .151]	.055 [.013; .097]	.052 [.024; .081]
$F_{r,70}$	.304 [.244; .365]	.285 [.224; .347]	.242 [.185; .299]	.219 [.176; .262]
$G_{30}$	.440 [.414; .458]	.384 [.332; .435]	.451 [.415; .488]	.451 [.431; .471]
$G_{70}$	.586 [.564; .608]	.529 [.469; .589]	.568 [.526; .610]	.589 [.568; .610]
$K_{k,f}$	.566 [.546; .585]	.502 [.441; .564]	.608 [.578; .639]	.601 [.580; .621]
$K_{k,t}$		.630 [.581; .679]		
$K_{u,f}$	.119 [.096; .142]	.028 [.000; .134]	.130 [.104; .156]	.127 [.102; .152]
$K_{u,t}$		.211 [.120; .305]		
**Experiment 3**				
$F_{n,25}$	0	0	.022 [.000; .065]	0
$F_{n,75}$			.050 [.000; .113]	
$F_{r,25}$	.117 [.072; .162]	.106 [.064; .147]	.098 [.057; .140]	.079 [.052; .106]
$F_{r,75}$	.349 [.297; .402]	.331 [.277; .385]	.296 [.242; .350]	.264 [.225; .304]
$G_{25}$	.420 [.400; .440]	.374 [.333; .416]	.407 [.367; .446]	.424 [.406; .442]
$G_{75}$	.588 [.568; .608]	.539 [.492; .586]	.561 [.519; .603]	.590 [.570; .609]
$K_{k,f}$	.512 [.493; .531]	.430 [.375; .485]	.575 [.545; .606]	.557 [.537; .578]
$K_{k,t}$		.600 [.557; .643]		
$K_{u,f}$	.104 [.083; .126]	.042 [.000; .123]	.115 [.090; .140]	.111 [.087; .135]
$K_{u,t}$		.167 [.089; .245]		

*Note.* Parameter estimates of fitted model variants of the KCM and FCM based on aggregated frequencies. 95% confidence intervals (CI) are shown in squared brackets

**Fig. 4:**
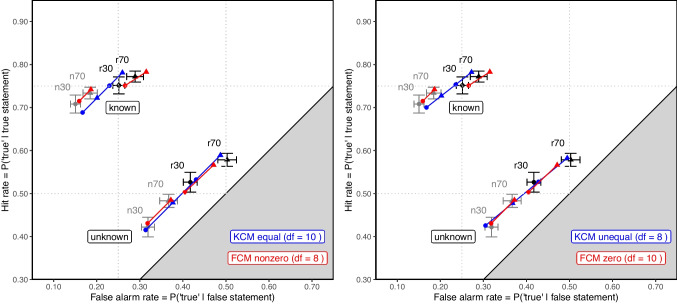
Observed and fitted false-alarm and hit rates of Experiment 1 in the ROC space. Observed frequencies are plotted in *grey* (new statements) and *black* (repeated statements) with error bars referring to $\pm 1$ SE. *Dots* and *triangles* correspond to base rates of 30% and 70% true statements, respectively. Model predictions and iso-sensitivity curves are shown in *blue* (KCM) and *red* (FCM). The left panel illustrates predictions of the model variants KCM_equal_ and FCM_nonzero_, whereas the right panel illustrates predictions of KCM_unequal_ and FCM_zero_. For improved readability, the *x*- and *y*-axes do not range from 0 to 1

### Results

Table 2 shows the average proportions of true judgments for all conditions. For our main analyses, we aggregated the frequencies of hits, false alarms, misses, and correct rejections across participants and statements for each of the $2\times 2\times 2 \times 2$ experimental conditions. We used the R package MPTinR (Singmann & Kellen, 2013) to analyze the aggregated frequencies. Model fit indices and parameter estimates are reported in detail in Tables 3 and 4, respectively.

Figure 4 illustrates the observed hit and false-alarm rates (gray and black points with error bars) in ROC space, as well as the predictions of the fitted model variants (red and blue points connected by lines). The two panels show different model versions of the KCM and the FCM. New and repeated statements are shown in gray and black, respectively, while dots and triangles represent base rates of 30% and 70%. All models showed a good overall fit, as indicated by relatively small deviations between observed frequencies and model predictions. While the KCM predicts that new and repeated statements lie on the same iso-sensitivity curve (blue line), the FCM predicts that new and repeated statements are located on different iso-sensitivity curves (red lines). The difference becomes particularly evident for known statements, irrespective of which specific model versions are considered.

Table 3 shows that the KCM_equal_, which resembles the original model version by Fazio et al. (2015), fitted the data well, $G^2(10) = 12.10$, $p =.278$, similar to the modified version KCM_unequal_, $G^2(8) = 7.91$, $p =.443$. Regarding the KCM_equal_, the knowledge manipulation had a substantial effect with $K_{k,t} = K_{k,f}=.521$ for known and $K_{u,t} = K_{u,f}=.102$ for unknown statements. In addition, the modified model version KCM_unequal_ indicated a difference in the knowledge parameters for true and false statements, $K_{k,t} =.569$ and $K_{k,f} =.449$; $K_{u,t} =.174$, and $K_{u,f} =.000$ (see Table 4). As expected, the base rate of true statements affected the estimated guessing parameter (KCM_equal_: $G_{30} =.349$ and $G_{70} =.420$; KCM_unequal_: $G_{30} =.305$ and $G_{70} =.368$). To assess the significance of these differences, we conducted nested model tests against restricted model versions assuming that guessing is equal in both base-rate conditions, $G_{30} = G_{70}$. For both the KCM_equal_ and the KCM_unequal_, the more restrictive model fitted the data significantly worse, $\Delta G^2(1) = 16.49$, $p <.001$ and $\Delta G^2(1) = 13.92$, $p <.001$, respectively. In line with the selective-influence assumption, the estimates for the *F* parameter were similar for the two base-rate conditions (KCM_equal_: $F_{r,30} =.200$ and $F_{r,70} =.212$; KCM_unequal_: $F_{r,30} =.177$ and $F_{r,70} =.201$) and did not differ significantly ($\Delta G^2(1) = 0.10$, $p =.746$ and $\Delta G^2(1) = 0.55$, $p =.460$, respectively).

The FCM_nonzero_, which resembles the original FCM most closely, fitted the data well, $G^2(8) = 9.17$, $p =.329$. Restricting the fluency parameter for new statements to zero, as assumed by the FCM_zero_, did not affect model fit, $G^2(10) = 9.17$, $p =.517$. Since the parameter estimates differed marginally between the two model versions, we focus on the FCM_zero_ in the following (for a detailed overview of parameter estimates, see Table 4). As expected, the knowledge manipulation had a substantial effect on the *K* parameter ($K_{k} =.556$ for known and $K_{u} =.113$ for unknown statements). The base-rate manipulation affected the guessing parameter as expected ($G_{30} =.358$, $G_{70} =.419$). Again, we conducted nested model tests by restricting guessing to be equal in both base-rate conditions, $G_{30} = G_{70}$. This led to a significantly worse model fit, $\Delta G^2(1) = 12.85$, $p = <.001$.

Interestingly, the $F_n$ parameters for new statements were estimated to be zero even in the model version FCM_nonzero_. The $F_r$ parameters for repeated statements differed slightly between base-rate manipulations, $F_{r,30} =.127$, $F_{r,70} =.158$. To assess whether base rates selectively influenced guessing but not repetition-related processes such as fluency, we tested whether the *F* parameters for repeated statements can be set equal across base-rate conditions, $F_{r,30} = F_{r,70}$. Conceptually, this resembles testing the interaction of base rate and repetition (Schmidt et al., 2025). The restricted model did not fit the data significantly worse, $\Delta G^2(1) = 1.52$, $p =.217$, indicating the selective influence of base rates on the guessing parameter.

A comparison of all KCM and FCM versions with the AIC and the BIC favored the FCM_zero_ (see Table 3). This result is in line with the ROC plot in Fig. 4, where the observed false-alarm and hit rates cannot be placed on a single, linear iso-sensitivity curve with unit slope as assumed by the KCM and the 2HTM. However, the amount of evidence for the FCM over the KCM is limited as indicated by the relatively small differences in AIC and BIC values. In addition, the KCM is preferred when focusing on the model versions with $\text {df}=8$ degrees of freedom.

**Table 5:** Fit indices for hierarchical MPT models

	Model	Base rate	PPP	WAIC
	Variant			
Experiment 1	KCM_equal_	70%	.196	4581.1
		30%	.402	
	KCM_unequal_	70%	.455	**4515.6**
		30%	.427	
	FCM_zero_	70%	.439	4591.8
		30%	.476	
Experiment 2	KCM_equal_	70%	.058	5533.9
		30%	.585	
	KCM_unequal_	70%	.497	**5505.5**
		30%	.588	
	FCM_zero_	70%	.330	5515.8
		30%	.188	
Experiment 3	KCM_equal_	75%	.022	6520.2
		25%	.347	
	KCM_unequal_	75%	.258	6462.6
		25%	.591	
	FCM_zero_	75%	.287	**6434.8**
		25%	.701	

*Note.* Separate hierarchical MPT models were fitted for each combination of model (KCM or FCM) and between-subject condition base rate of true statements (70% or 30% in Experiment 1 and 2, 75% and 25% in Experiment 3). Posterior predictive *p* values (PPP) were computed for each fitted model separately, whereas the Watanabe–Akaike information criterion (WAIC) was computed for each model across base-rate conditions

As a robustness check, we also performed model selection for hierarchical latent-trait MPT models fitted to the individual false-alarm and hit rates (Klauer, 2010) using the R package TreeBUGS (Heck et al., 2018). We ensured convergence by fitting all models five times with 50,000 MCMC iterations each. We performed model selection with the widely applicable information criterion (WAIC). To evaluate overall model performance, we summed the WAIC values across the two base-rate conditions. Similar to AIC and BIC, we cannot interpret absolute WAIC values but only the difference in WAIC values between different models. To assess absolute model fit, we also computed posterior predictive *p* values (PPP; Klauer, 2010). Table 5 shows that all hierarchical KCM and FCM versions fitted well, as indicated by PPP values above the commonly used threshold of .05 (all $p\text {~values} >.196$). Regarding the WAIC values, the KCM_equal_ was slightly favored over the FCM_zero_ with a WAIC difference of 10.7. The KCM_unequal_ with unequal knowledge parameters performed best with a WAIC value 65.5 points lower.

### Discussion

Overall, both the KCM and the FCM fit the data well, with the FCM being slightly preferred by several information criteria. The relatively similar performance of all model versions emphasizes the challenge of providing a critical test for the two competing accounts. The base-rate manipulation of truth judgments did not result in a clear misfit of any model version. Figure 4 also shows that informing participants about the base rate of true statements had a relatively small effect on truth judgments, especially for known statements.

## Experiment 2

Experiment 2 serves as a preregistered, more critical test of the KCM and the FCM.^2^ Since Experiment 1 did not provide clear evidence for either model, we aimed for a stronger effect of the base-rate manipulation. A simulation-based power analysis (see below) indicated that a larger effect increases the chances of detecting model misfit of both the KCM and the FCM. To increase the effect of the different base rates, participants in Experiment 2 received a small monetary incentive for correct judgments. Providing monetary rewards for correct judgments is a common approach to incentivize response patterns in line with the base rate (Malejka & Bröder, 2019). Participants received one point for each correct judgment, resulting in a maximum additional reward of 50% relative to the default participation fee. Moreover, base-rate consistent responding was incentivized by giving feedback on the percentage of correct judgments after every 12 statements.

**Fig. 5:**
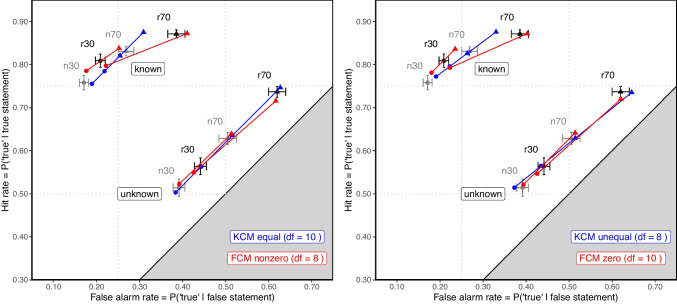
Observed and fitted false-alarm and hit rates of Experiment 2 in ROC space. Observed frequencies are plotted in *grey* (new statements) and *black* (repeated statements) with error bars referring to $\pm 1$ SE. *Dots* and *triangles* correspond to base rates of 30% and 70% true statements, respectively. Model predictions and iso-sensitivity curves are shown in *blue* (KCM) and *red* (FCM). The left panel illustrates predictions of the model variants KCM_equal_ and FCM_nonzero_, whereas the right panel illustrates predictions of KCM_unequal_ and FCM_zero_. For improved readability, the *x*- and *y*-axes do not range from 0 to 1

### Methods

#### Power analysis

To assess the statistical power to detect model misfit of the KCM and the FCM in a likelihood ratio test, we simulated data sets for each the KCM and the FCM using the parameter estimates from Experiment 1. Based on 10,000 simulated data sets, we estimated the power of detecting model misfit by fitting both the KCM_equal_ and the FCM_zero_ to datasets simulated by each model. We chose a relatively low value for the type I error rate ($\alpha = 1\%$) to reduce the risk of rejecting a model merely due to the relatively large sample sizes in our study. For the preregistered sample size ($N = 175$), the power to detect model misfit was around 90% for both models (i.e., $1 - \beta = 89.3\%$ with data simulated from the KCM_equal_ and $1 - \beta = 90.8\%$ with data simulated from the FCM_zero_).^3^ The actual sample size ($N = 208$, see below) was larger than specified in the a priori power analysis, which corresponds to a power of 95.0% and 96.7%.

#### Participants

We recruited 249 native German speakers through the panel provider *bilendi*. To prevent participants from providing overly fast responses, each statement was presented for two seconds before participants could provide a judgment (see Procedure below). Hence, in contrast to Experiment 1, we did not exclude participants if they rated 25% of statements extremely fast. We monitored whether participants switched the browser window more than five times to discourage them from looking up correct answers to the statements, but this did not result in the exclusion of any participants. Besides these changes, we applied the same exclusion criteria as in Experiment 1. We excluded 26 participants who did not complete the study, two participants who indicated not to have worked on the study seriously, one participant who exceeded the limit of 30 s for truth judgments in 25% of the trials, ten participants who finished the study in less than 10 min, and two participants due to technical issues that prevented the recording of response times. On the trial level, we excluded 20 extremely fast (< 2.2 s) judgments and 74 extremely slow (> 30 s) judgments.^4^ The final sample comprised $N = 208$ participants (100 male, 107 female, one other) with a mean age of 48.1 ($SD = 15.4$, ranging from 18 to 75) and an average of 71.55 truth judgments per participant.

#### Design, materials & procedure

The design and materials were mostly identical to Experiment 1, while participants received a small monetary incentive for correctly judging the truth of statements. To ensure that participants were aware of the incentive structure, participants rated two example statements as well as an additional filler statement before the actual judgment phase. For each example, participants directly received feedback on whether they would have received a point. In the actual judgment phase, participants received feedback on their current accuracy (both the absolute number and the current percentage of correct judgments) after every block of 12 statements. As a minor change to Experiment 1, each statement was displayed for 2.2 s before a judgment could be made to discourage participants from judging statements overly fast.

### Results

The mean percentage of true judgments in Table 2 and the ROC plot in Fig. 5 show that the base-rate manipulation had a stronger effect on truth judgments than in Experiment 1. Visually, this is represented by a greater distance between the observed hit and false-alarm rates of the 30% and 70% base-rate conditions. Note that, both for known and unknown statements, the hit and false-alarm rates for new statements in the 70% base-rate condition were higher than those for repeated statements in the 30% base-rate condition, resulting in a different order of the $2\times 2$ conditions relative to Experiment 1. Unlike the KCM, the FCM posits distinct iso-sensitivity curves for new and repeated statements, an effect especially pronounced for known statements (red lines).

The KCM_equal_ did not fit the data well, $G^2(10) = 25.32$, $p =.005$. According to the preregistered significance level of $\alpha =1\%$, the less restrictive KCM_unequal_ could not be rejected, $G^2(8) = 18.87$, $p =.016$. Thus, we focus on the parameter estimates of the KCM_unequal_ in the following. The knowledge manipulation had a large effect, while the knowledge parameters also differed between true and false statements ($K_{k,t} =.630$, $K_{k,f} =.502$, $K_{u,t} =.211$, and $K_{u,f} =.028$). As expected, the base rate of true statements had a significant effect on the guessing parameter, $G_{30} =.384$ and $G_{70} =.529$ ($\Delta G^2(1) = 78.04$, $p <.001$). However, the base-rate manipulation also affected the *F* parameter for repeated statements, $F_{r,30} =.104$ and $F_{r,70} =.285$ ($\Delta G^2(1) = 22.21$, $p <.001$), indicating a lack of selective influence.^5^

The FCM_nonzero_ fit the data well, $G^2(8) = 13.14$, $p =.107$, as did the FCM_zero_, $G^2(10) = 14.84$, $p =.138$. Table 4 shows that parameter estimates were almost identical for both model versions since the two *F* parameters for new statements were estimated to be approximately zero. Hence, we focus on the results of the simplified model version FCM_zero_. As expected, the base-rate manipulation affected the guessing parameter, $G_{30} =.451$ and $G_{70} =.589$ ($\Delta G^2(1) = 80.09$, $p <.001$). However, the *F* parameters for repeated statements also significantly differed between base-rate conditions, $F_{r,30} = .052$ and $F_{r,70} = .219$ ($\Delta$ $G^2(1) = 39.76$, $p < .001$), implying that the base-rate manipulation did not selectively influence the guessing parameter.

Table 3 shows that the FCM models were also preferred by the AIC and BIC, with the restricted version FCM_zero_ having the smallest values. Correspondingly, in Fig. 5, the observed hit and false-alarm rates are more in line with the predictions of the FCM. For known statements, the FCM assumes two distinct iso-sensitivity lines for new and repeated statements. For unknown statements, both models make very similar predictions with the four hit and false-alarm rates following a single, linear iso-sensitivity curve.

Again, we fitted hierarchical MPT models as a robustness check. Table 5 shows that the PPP fit indices were above the common threshold of .05 for all models. According to the WAIC, FCM_zero_ was preferred over KCM_equal_ with a difference of 18.1, whereas the modified model KCM_unequal_ performed best with a WAIC difference of 10.3.

### Discussion

Experiment 2 implemented a stronger base-rate manipulation by providing small monetary incentives for correct judgments and intermediate feedback on accuracy. In addition, the larger sample size ($N = 208$ compared to $N = 170$ in Experiment 1) ensured a higher power for testing both models. These improvements facilitated the detection of differences in model fit between the KCM and the FCM. For the aggregated data, the original version of the KCM did not fit, and the FCM performed best. A modified version of the KCM with different knowledge parameters for true and false statements performed better and was preferred over the FCM in the hierarchical analysis. The results thus provide mixed evidence for the assumption that the repetition-based truth effect does not reflect merely a response bias (as assumed by the KCM) but a decrease in discrimination performance (as assumed by the FCM). However, the base-rate manipulation did not only influence the guessing parameter *G* but also the parameter *F*, both in the KCM and the FCM. This is a challenging finding since it questions the interpretation of the *F* parameter as the relative increase in processing fluency due to repetition.

## Experiment 3

Given the mixed evidence in the previous studies, Experiment 3 aimed at an even stronger base-rate manipulation to facilitate the statistical discrimination between both models. To do so, we communicated base rates of true statements to participants that were slightly more biased (i.e., 75% and 25% instead of 70% and 30%) and could be represented as simple ratios for a more intuitive understanding (e.g., “on average, one out of four statements is true”). However, to maintain high statistical power, the actual ratio of true statements was identical to Experiments 1 and 2. We consider the discrepancies between the communicated and the actual base rates to be unproblematic because they are rather small and unlikely to be detected by the participants in light of the difficulty of the statements. Additionally, we also increased the monetary incentives for correct judgments by allowing participants to receive an additional reward of up to 150% of their default participation fee. Experiment 3 was again preregistered based on the results of a simulation-based power analysis.^6^

### Methods

#### Power analysis

For the simulation-based power analysis, we used the parameter estimates obtained in Experiment 2 as data-generating values. We again chose a significance level of $\alpha =1\%$. The power to detect model misfit for a sample size of $N = 225$ was above 95% for all models ($1 - \beta = 97.0\%$ for data simulated from the KCM_equal_ and $1 - \beta = 98.8\%$ for data simulated from the FCM_zero_).^7^ The power for the actual sample size ($N=240$) was estimated to be 97.9% and 99.3%.

#### Participants

We recruited 281 German native speakers via the panel provider *bilendi* and applied the same filter criteria as in Experiment 2. We excluded 30 participants who did not complete the entire study, four participants showing overly fast completion times (total time < 10 min) and seven participants due to technical issues preventing the recording of response times. The exclusion criteria were whether participants exceeded the limit of 30 s for truth judgments in 25% of the trials, the question of whether participants worked seriously on the study and the monitoring of changes of the browser window did not lead to any further exclusions. We excluded 41 judgments with extremely fast (< 2.2 s) or extremely slow (> 30 s) response times.^8^. The final sample consisted of $N = 240$ participants (108 male, 131 female, one other) with a mean age of 49.0 ($SD = 15.1$, ranging from 18 to 75) and an average of 71.83 truth judgments per person.

#### Design, materials, & procedure

The design and materials were identical to Experiments 1 and 2. However, the base rate of true statements communicated to the participants in the instructions of the truth-judgment phase was either 75% or 25%, while the actual base rate of true statements was still 48 or 24 out of 72. Moreover, we tripled the monetary reward for correct judgments. Thereby, participants could gain up to 150% of their default participation fee.

### Results

**Fig. 6:**
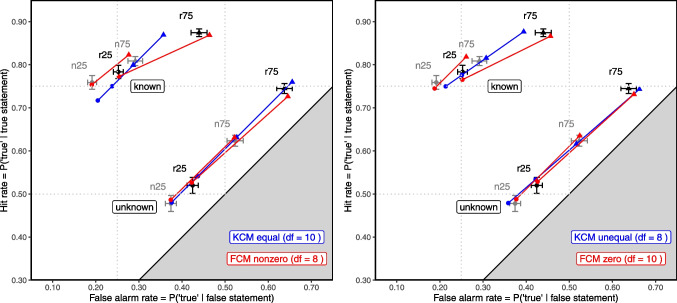
Observed and fitted false-alarm and hit rates of Experiment 3 in the ROC space. Observed frequencies are plotted in *grey* (new statements) and *black* (repeated statements) with error bars referring to $\pm 1$ SE. *Dots* and *triangles* correspond to base rates of 25% and 75% true statements, respectively. Model predictions and iso-sensitivity curves are shown in *blue* (KCM) and *red* (FCM). The left panel illustrates predictions of the model variants KCM_equal_ and FCM_nonzero_, whereas the right panel illustrates predictions of KCM_unequal_ and FCM_zero_. For improved readability, the *x*- and *y*-axes do not range from 0 to 1

Table 2 and the ROC plot in Fig. 6 show that the observed hit and false-alarm rates in Experiment 3 were very similar to those in Experiment 2. Model predictions suggest a slightly superior fit of the FCM than the KCM. For known statements, the observed hit and false-alarm rates were better represented by two separate iso-sensitivity curves as assumed by the FCM. For unknown statements, hit and false-alarm rates were aligned on a straight line approximately parallel to the main diagonal, a pattern in line with both models.

The KCM_equal_ model did not fit the data well, $G^2(10) = 41.34$, $p <.001$, whereas the less restrictive KCM_unequal_ was not rejected at the preregistered significance level of $\alpha =1\%$, $G^2(8) = 16.79$, $p =.032$. While both models assume different knowledge parameters for known and unknown statements, only the KCM_unequal_ can account for the finding that knowledge differed between true and false statements ($K_{k,t} =.600$, $K_{k,f} =.430$, $K_{u,t} =.167$, and $K_{u,f} =.042$). In the following, we thus focus on the parameters of the KCM_unequal_. As expected, the base rate of true statements affected guessing, $G_{25} =.374$ and $G_{75} =.539$ ($\Delta G^2(1) = 123.88$, $p <.001$). However, the base rate also affected the *F* parameter, $F_{r,25} =.106$ and $F_{r,75} =.331$ ($\Delta G^2(1) = 43.37$, $p <.001$),indicating a lack of selective influence.

Both versions of the FCM fit the data well (FCM_nonzero_: $G^2(8) = 8.79$, $p = .361$; FCM_zero_: $G^2(10) = 11.31$, $p = .334$). The parameter estimates of both models were very similar, as the $F_n$ parameter for new statements was estimated to be approximately zero. In the following, we thus focus on the parameter estimates of FCM_zero_. The base rate of true statements affected guessing as expected, $G_{25} = .424$ and $G_{75} = .590$ ($\Delta G^2(1) = 138.72$, $p < .001$). However, the base rate also had a significant effect on the *F* parameter for repeated statements, $F_{r,25} = .079$ and $F_{r,75} = .264$ ($\Delta$ $G^2(1) = 55.61$, $p < .001$), implying a violation of selective influence.

Replicating the results of Experiment 2, the FCM models were preferred by the AIC and BIC (see Table 3). Again, the FCM_zero_ showed the smallest values. This is also visible in the ROC space in Fig. 6 since the observed rates of hits and false alarms display the smallest deviations from the model predictions. As a robustness check for model selection, we again fitted hierarchical MPT models for individual response frequencies. Table 5 shows that, according to the commonly used threshold of .05 for PPP values, the absolute fit of the models FCM_zero_ and KCM_unequal_ was satisfactory. In contrast, the KCM_equal_ with equal knowledge parameters, resembling the original KCM, fitted well in the 25% base-rate condition but worse in the 75% condition. The WAIC favored the FCM_zero_ over the KCM_equal_ with a difference of 85.4. In addition, the KCM_unequal_ performed better than the original KCM_equal_ with a WAIC difference of 57.6, but worse than the original FCM with a WAIC difference of 27.8.

### Discussion

Experiment 3 provided a stronger base-rate manipulation by communicating slightly more extreme base rates (75% and 25%) and increasing the monetary incentives for correct judgments. Besides these changes in the design, the power for differentiating between the KCM and FCM also increased due to the larger sample size. The results provide less ambiguous results than Experiment 2. Both versions of the FCM showed a good model fit, whereas the original version of the KCM did not fit the data. The KCM version with separate knowledge parameters for true and false statements performed better, but not as well as the FCM. Unexpectedly, base rates not only influenced the guessing parameter *G* but also the *F* parameter, which is thought to reflect increased processing fluency due to repetition.

## General discussion

We tested two accounts of the repetition-based truth effect, the knowledge-condition model (KCM) and the fluency-conditional model (FCM; Fazio et al., 2015). Both models assume that knowledge, fluency, and guessing are cognitive processes relevant for judging truth. The KCM assumes that repetition affects truth judgments only when the correct answer is unknown, implying that the truth effect reflects a response bias. In contrast, the FCM assumes that repetition has a dominant effect with a certain unconditional probability *F* irrespective of whether knowledge is available. As a result, the model predicts a decrease in discrimination performance in distinguishing between true and false repeated statements. The FCM implies the somewhat counterintuitive finding that people do not necessarily rely on knowledge if possible. Rather, metacognitive cues such as processing fluency are used even in the presence of knowledge.

We assessed predicted ROC curves and highlighted important substantive and technical assumptions of both models. Based on these considerations, we derived adapted model versions that ensure model identifiability (e.g., for the KCM, by constraining the *F* parameter for new items to be zero) and relax critical assumptions (by fitting a modified KCM model with separate knowledge parameters for true and false statements). As a validation and stronger test of the models, we performed three studies with an extended experimental design by manipulating the base rate of true statements as a between-subjects factor.

Experiment 1 informed participants that the base rate of true statements was either 70% or 30% but did not provide any monetary incentives for correct judgments. Accordingly, the base-rate manipulation had only a small effect on response behavior, and both the KCM and FCM fitted the data well. The FCM with parameter *F* restricted to zero for new statements was preferred when analyzing aggregated data, while the adapted KCM with separate knowledge parameters was preferred in the hierarchical analysis.

Experiment 2 provided a small monetary incentive for correct judgments and feedback after each block of 12 trials. This resulted in a larger effect of the base rate, and in turn, larger discrepancies in model fit. The two versions of FCM were preferred when analyzing aggregated response frequencies. The original KCM failed to account for the data, whereas a modified KCM with separate knowledge parameters for true and false statements was not rejected and performed best in the hierarchical analysis.

Experiment 3 communicated slightly more extreme base rates (75% and 25%) to participants and provided larger monetary incentives for correct judgments. The results unambiguously favored the FCM model versions over all KCM model versions.

Across all three experiments, the FCM fitted the data well and was preferred over the original version of the KCM, which assumes equal knowledge for true and false statements. These findings support the notion that the repetition-based truth effect may not merely reflect a response bias conditional on the absence of knowledge, but rather results in a decreased performance to discriminate between true and false statements (Fazio et al., 2015, 2019). Thus, our results provide cautious support for the conclusion of Fazio et al. (2015) and Calio et al. (2020) that cognitive processes affected by repeated exposure, such as increased fluency, familiarity, or related processes, dominate truth judgments even when knowledge is available. However, a new version of the KCM allowing for different knowledge of true and false statements could not be rejected and even outperformed the FCM when the base-rate manipulation was relatively weak (i.e., in Experiment 1 and partly in Experiment 2). Hence, conceptualizing the repetition-based truth effect as a response bias may also explain truth judgments fairly well under certain boundary conditions. Although our experiments provided high statistical power for detecting model misfit for the KCM and FCM, it remains challenging to clearly distinguish the two models empirically. Future research should consider the modified KCM version as a competing account that cannot be rejected yet.

For both the FCM and the KCM, the base-rate manipulation affected not only the guessing parameter *G* but also the *F* parameter for repeated statements. This lack of selective influence is problematic for interpreting the *F* parameter because it is not obvious why different base rates would affect the probability of relying on processing fluency when judging repeated statements. Thus, it is difficult to attribute the contribution of the *F* parameter to the repetition-based truth effect to a reliance on processing fluency. The interpretation of the *F* parameter generally remains ambiguous, because it can represent other processes that may cause repeated statements to be judged as more true, such as recognition, familiarity, or coherence (Unkelbach et al., 2019). To clarify the interpretation of the *F* parameter, future validation studies are required, which may test the FCM and the KCM by manipulating other factors besides repetition that have been hypothesized to increase processing fluency (e.g., visual contrast; Reber and Schwarz, 1999, but see Aktepe and Heck, 2025a). Moreover, future research should investigate whether different theoretical accounts of the truth effect (Unkelbach et al., 2019) are better aligned with the FCM or the KCM (i.e., whether the effect of repetition is assumed to be conditional on the absence of knowledge or not). Thereby, multinomial modeling may contribute to a more precise specification and testing of competing theories.

Our theoretical analysis showed that assessing predictions for truth judgments in ROC space offers valuable insights into underlying assumptions and implications of competing models (Malmberg, 2002). Assessing predicted and fitted ROC curves enables researchers to assess the validity of auxiliary model assumptions, which may have important substantive consequences. For instance, assuming that people have equal knowledge of true and false statements implies an iso-sensitivity curve with a unit slope, whereas weakening this assumption allows for different slopes (Hilbig, 2012; Malejka & Bröder, 2019). In MPT modeling, researchers should generally check model identifiability and consider multiple model versions with different auxiliary assumptions (Schmidt et al., 2025), which was particularly important for the KCM in the present case. A formal modeling perspective also highlights the relevance of auxiliary assumptions. Concerning the assumption of equal knowledge for true and false statements in the KCM, one may either optimize the experimental design by matching the materials carefully or modify the model by loosening the constraint.

From an applied perspective, the competing models provide different conceptualizations of the cognitive processes underlying truth judgments, suggesting different interventions in the context of misinformation. If the truth effect resembles a response bias, as assumed by the KCM, interventions could improve people’s background knowledge as a protective factor or provide people with incentives for making accurate judgments (Brashier et al., 2020), as done in the present study. In contrast, when conceptualizing the truth effect as a general reduction in discrimination performance, as assumed by the FCM, people may profit more from other interventions, such as warnings or inoculations (Nadarevic & Aßfalg, 2017; van der Linden et al., 2017).

We did not test for the existence of qualitative individual differences in cognitive processing, which could be moderated by age or traits such as need for cognition (De keersmaecker et al., 2020). Brashier et al. (2017) showed that older adults rely more heavily on prior knowledge and are thus more resistant to the truth effect for known falsehoods – an outcome consistent with the KCM but not with the FCM. Such age-related differences imply that the relative fit of the two models may vary across individuals, offering a promising direction for future research. The present approach can be extended by fitting a hierarchical latent-class model (Bartlema et al., 2014), assuming that different participants are better described either by the KCM or the FCM, with age serving as a predictor of group membership. Identifying relevant moderators could advance theoretical understanding and inform applied interventions aimed at mitigating susceptibility to misinformation in different populations.

### Signal detection theory as an alternative modeling framework

Signal detection theory (SDT) offers an alternative framework for modeling truth judgments (Unkelbach, 2007; Gawronski et al., 2023; Batailler et al., 2022). According to SDT, judgments are based on a latent, continuous signal (e.g., the feeling or perception of truth), which has to be transformed into a dichotomous “true” or “false” response (Aktepe & Heck, 2025b). SDT models for truth judgments assume two parameters: First, truth sensitivity $d'$ refers to the amount of knowledge about a set of statements and is defined as the location of the signal distribution relative to the noise distribution. Second, the response bias *c* refers to the general tendency to give a “true” response irrespective of a statement’s content and represents the threshold on the latent continuum above which a statement is judged as true.

Unkelbach (2007) proposed an SDT model for the repetition-based truth effect wherein fluency was defined as an experience, whereas familiarity was considered to be an interpretation of the fluency experience. This theoretical conceptualization underscores the close relationship of constructs such as familiarity and fluency. Importantly, Unkelbach (2007) assumed that the truth effect represents a differential response bias for repeated and new statements due to differences in fluency. This interpretation closely aligns with the KCM and the 2HTM. However, an empirical validation of Unkelbach’s assumption remains elusive, as the reported analyses primarily relied on the estimated SDT parameters without testing model fit or performing model selection.

SDT closely resembles the KCM and the 2HTM in conceptualizing the truth effect as a response bias, but the underlying model assumptions differ. Whereas SDT assumes a continuous latent evidence signal, MPT models such as the KCM and the 2HTM assume a finite number of discrete latent states (Malmberg, 2002). As a result, SDT does not predict straight ROC lines as in Fig. 2, but rather nonlinear iso-sensitivity curves. The literature on recognition memory shows that empirical tests between SDT and 2HTM models are difficult to conceive, require several auxiliary assumptions, and often result in mixed evidence (Klauer & Kellen, 2011). In many cases, however, the two modeling frameworks provide convergent conclusions, implying that either model can provide valid estimates of knowledge and response bias (Batchelder & Alexander, 2013). However, MPT modeling is required for testing the FCM model since it does not have a direct equivalent in the SDT framework.

### Conclusion

Our experiments demonstrated the dominance of repetition over knowledge in the repetition-based truth effect. Through our experiments, the conceptualization of the repetition-based truth effect as a reduced discrimination performance was supported. However, when allowing for different knowledge for true and false statements, one cannot reject the alternative account that the truth effect is merely a response bias conditionally on the absence of knowledge. A major limitation of all tested models concerns the interpretation of the *F* parameter as a measure of processing fluency. Given that the base-rate manipulation not only influenced the guessing parameter *G* but also the parameter *F*, further validation of the competing multinomial models is vital to test the models’ substantive interpretation.

## Data Availability

All data and analysis scripts are available at the Open Science Framework (OSF): https://osf.io/c86wq/.

## Appendix: Derivation of ROC curves

The following formulas demonstrate how to derive ROC curves based on the model equations of the FCM Eq. 1 and Eq. 2

1 $$\begin{aligned} P(\text {Hit})&= F + (1-F)\cdot K + (1-F)\cdot (1-K) \cdot G\end{aligned}$$

2 $$\begin{aligned} P(\text {FA})&= F + (1-F)\cdot (1-K) \cdot G. \end{aligned}$$

First, we solve the Eq. 2 for G

3 $$\begin{aligned} G = \frac{P(\text {FA}) - F}{(1-F)\cdot (1-K)}. \end{aligned}$$

As a result, it is possible to simplify the first part of the model Eq. 1 resulting in

4 $$\begin{aligned} P(\text {Hit})&= (1-F)\cdot K + P(\text {FA}). \end{aligned}$$

## Footnotes

1 As explained in the next section, the fitted KCM also included the constraint $F_n =0$, overall resulting in $\text {df}=2$ degrees of freedom. The corresponding analysis file is available at the OSF.

2 The preregistration is available at https://aspredicted.org/9wjz-tnfx.pdf. We also preregistered to fit mixed-effects logistic regressions, which are irrelevant for the MPT model comparison, and are thus only reported in the supplementary materials.

3 The estimated power slightly differs from the preregistration since we based the power analysis on different variants of the KCM and FCM.

4 The exclusion of overly fast trials was not preregistered, as the procedure should have ensured a minimum time for responding. However, due to technical issues, we still had to exclude overly fast responses.

5 We also preregistered testing the equality $s_{G,30n} = s_{G,30r}$ within the 2HTM, a model that is equivalent to the KCM but differs in parameterization. We do not report this test since fitting the 2HTM was omitted for simplicity.

6 The preregistration is available at https://aspredicted.org/pzwm-2vp3.pdf. The preregistered mixed-effects logistic regression analyses are reported in the supplementary materials.

7 The estimated power slightly differs from the preregistration since we based the power analysis on different variants of the KCM and FCM.

8 The exclusion of overly fast trials was not preregistered, as the procedure should have ensured a minimum time for responding. However, due to technical issues, we still excluded overly fast responses

## Code availability

All data and analysis scripts are available at the Open Science Framework (OSF): https://osf.io/c86wq/.

## Declarations

### Open practices statement

Experiments 2 and 3 were preregistered, and the corresponding preregistration files can be found at https://aspredicted.org/9wjz-tnfx.pdf (Experiment 2) and https://aspredicted.org/pzwm-2vp3.pdf (Experiment 3).
